# The bucket test differentiates patients with MRI confirmed brainstem/cerebellar lesions from patients having migraine and dizziness alone

**DOI:** 10.1186/s12883-019-1442-z

**Published:** 2019-09-03

**Authors:** Tzu-Pu Chang, Ariel A. Winnick, Yung-Chu Hsu, Pi-Yu Sung, Michael C. Schubert

**Affiliations:** 1 Department of Neurology/Neuro-medical Scientific Center, Taichung Tzu Chi Hospital, Buddhist Tzu Chi Medical Foundation, Taichung City, Taiwan; 2 Department of Neurology, Tzu Chi University, Hualien, Taiwan; 3 Soroka University Hospital and Faculty of Health Sciences, Ben-Gurion University of the Negev, Beer-Sheva, Israel; 4 Division of Neurology, Department of Internal Medicine, Ditmanson Medical Foundation Chia-Yi Christian Hospital, Chiayi, Taiwan; 5 Department of Physical Medicine and Rehabilitation, Taichung Tzu Chi Hospital, Buddhist Tzu Chi Medical Foundation, Taichung City, Taiwan; 6 Laboratory of Vestibular NeuroAdaptation, Department of Otolaryngology - Head and Neck Surgery, Johns Hopkins University, Baltimore, MD 21205 USA; 7 Department of Physical Medicine and Rehabilitation, Johns Hopkins University, Baltimore, MD 21205 USA

**Keywords:** Vestibular migraine, Central vestibular disorder, Subjective visual vertical (SVV), Bucket test, Nystagmus

## Abstract

**Background:**

Amongst the most challenging diagnostic dilemmas managing patients with vestibular symptoms (i.e. vertigo, nausea, imbalance) is differentiating dangerous central vestibular disorders from benign causes. Migraine has long been recognized as one of the most common causes of vestibular symptoms, but the clinical hallmarks of vestibular migraine are notoriously inconsistent and thus the diagnosis is difficult to confirm. Here we conducted a prospective study investigating the sensitivity and specificity of combining standard vestibular and neurological examinations to determine how well central vestibular disorders (CVD) were distinguishable from vestibular migraine (VM).

**Method:**

Twenty-seven symptomatic patients diagnosed with CVD and 36 symptomatic patients with VM underwent brain imaging and clinical assessments including; 1) SVV bucket test, 2) ABCD^2^, 3) headache/vertigo history, 4) presence of focal neurological signs, 5) nystagmus, and 6) clinical head impulse testing.

**Results:**

Mean absolute SVV deviations measured by bucket testing in CVD and VM were 4.8 ± 4.1° and 0.7 ± 1.0°, respectively. The abnormal rate of SVV deviations (> 2.3°) in CVD was significantly higher than VM (*p* < 0.001). Using the bucket test alone to differentiate CVD from VM, sensitivity was 74.1%, specificity 91.7%, positive likelihood ratio (LR+) 8.9, and negative likelihood ratio (LR-) 0.3. However, when we combined the SVV results with the clinical exam assessing gaze stability (nystagmus) with an abnormal focal neurological exam, the sensitivity (92.6%) and specificity (88.9%) were optimized (LR+ (8.3), LR- (0.08)).

**Conclusion:**

The SVV bucket test is a useful clinical test to distinguish CVD from VM, particularly when interpreted along with the results of a focal neurological exam and clinical exam for nystagmus.

**Electronic supplementary material:**

The online version of this article (10.1186/s12883-019-1442-z) contains supplementary material, which is available to authorized users.

## Background

Amongst the most challenging diagnostic dilemmas, managing patients with vestibular symptoms (i.e. vertigo, nausea, imbalance) is differentiating dangerous central vestibular disorder (CVD) from benign causes. While numerous scientific articles have discussed how to differentiate CVD from peripheral vestibulopathy [1–3], few studies explore the differential diagnosis between dangerous CVD and vestibular migraine (VM). VM is common and benign though regarded as a broad-spectrum central disorder [4]. Migraine has long been recognized as one of the most common causes of vestibular symptoms, but the clinical hallmarks of VM are notoriously inconsistent and thus the diagnosis is difficult to confirm [5, 6]. The current diagnostic criteria of VM are 1) episodes of recurrent spontaneous vertigo of moderate to severe degree, 2) personal history of migraine fulfilling the criteria of the International Headache Society (IHS), and 3) the accompaniment of migraine features during vertigo attacks [7]. Despite the recently adopted clinical definition of VM, the clinical and oculographic evidence is varied. For example, although most head impulse testing is normal in patients with VM, patients with VM can have abnormal head impulse or abnormal caloric examination suggesting a peripheral cause [8]. Abnormal ocular motor function has been reported in VM suggesting a central cause yet as many as 35% of patients with VM are unable to be classified as either having a central or peripheral origin [8–10]. Finally, low velocity nystagmus that does not follow the expected characteristics of a peripheral etiology (i.e. downbeat nystagmus instead of persistent horizontally directed nystagmus) is common in patients with VM [11], yet this finding, unfortunately, causes a majority of patients with VM to be diagnosed as having a CVD. Although migraine is a pathophysiology involving the cortical regions that process vestibular afference, it is not considered as sinister as CVD. In addition to the expectation of a better clinical outcome, the differences in acute treatment and the long-term prophylaxis are completely different between migraine and dangerous central lesions. Therefore, the prompt differentiating of CVD from VM is a critical goal for clinicians. Despite this, the importance of differentiating CVD from VM is overlooked for a number of reasons.

First, VM is thought to be easily differentiated from CVD based on a history that includes the presence of migraine headache and associated symptoms (i.e., photophobia and phonophobia). However, in roughly half of patients with VM, the vestibular symptoms are not accompanied by headache [6]. Additionally, most strokes caused by vertebral artery dissection and 14% of transient ischemic attacks (TIA) in the vertebrobasilar territories also present with headache, further complicating the differential diagnosis [12, 13]. Finally, while the presence of photophobia and phonophobia can sometimes be useful, they are non-specific, particularly in the acute stage of vertigo related to migraine [14].

Second, VM is supposed to be recurrent (i.e., episodic vestibular syndrome), different from CVD that presents as a monophasic prolonged vertigo (i.e., acute vestibular syndrome). Nonetheless, vertebrobasilar TIA can cause recurrent bouts of dizziness, which may be confused with VM symptoms [15]. In addition, duration of VM episodes are notoriously variable, where intense episodes may occur on the order of minutes to hours to days [9], or as constant and lingering symptoms of less intensity but lasting months or years [16]. Recently, emerging evidence confirms vestibular symptoms from vestibular migraine can to be chronic [17]. The initial episodes of VM can be frighteningly similar with the acute vestibular syndrome or an acute but transient vestibular syndrome (< 24 h).

Third, CVD is traditionally thought be easily diagnosed on the identification of focal neurological deficits, however, recent studies report many patients with CVD have no focal neurological signs [18–21]. In the absence of an abnormal imaging study, patients with CVD and a normal focal neurological exam are often misdiagnosed as having an unspecified peripheral vestibular disorder [22].

Fourth, some specialists expect the three-step examination that includes the head impulse test, evaluation of nystagmus, and test of skew deviation (HINTS) can differentiate CVD from all benign vertigo. Indeed, when applied for differentiating CVD from peripheral disorders in the acute vestibular syndrome, HINTS is very useful with an optimal sensitivity (100%) and specificity (96%) [1]. However, it is clinically inappropriate to use HINTS to differentiate CVD from VM since the head impulse test is usually normal in both CVD and VM.

For these reasons, we believe that developing a bedside diagnostic battery to differentiate dangerous CVD from VM is of a clinical significance. We examined how well a series of clinical bedside tests and medical histories distinguished patients with CVD from patients with VM. Our goal was to identify any combination of neuro-vestibular bedside exams that might improve the sensitivity and specificity to distinguish CVD from VM.

## Method

### Patient subjects and healthy controls

We prospectively consented 66 consecutive and symptomatic patients diagnosed with CVD (*n* = 27) or VM (*n* = 36) from the Taichung Tzu Chi Hospital Neurology Dizziness Clinic in Taiwan between January 1, 2013 and December 31, 2013. Healthy volunteers with age and gender matched with CVD were recruited to be the healthy controls (*n* = 27). All the patients who had prolonged vestibular symptoms (≧ 24 h) without clear diagnoses underwent brain MRI. Patients with CVD were diagnosed by the results of brain MRI and had verified brainstem and/or cerebellar lesions confirmed by neuroradiologists. Patients with VM were diagnosed using the accepted diagnostic criteria of VM as published in the International Classification of Vestibular Disorders (ICVD) (Additional file 2: Table S1) [7]. Each of the 63 patients were experiencing vertigo, dizziness or unsteadiness at the time of their clinical exam.

Any patient with history of a peripheral vestibular disorder (i.e. BPPV or vestibular hypofunction) was excluded. In the VM group, those with a history of structural brain lesions were excluded. In the CVD group, any patient with a preceding history of migraine was excluded. Considering the effect of vestibular compensation, we also excluded the patients diagnosed with brainstem or cerebellar tumors. Healthy controls were excluded if they reported vestibular symptoms. MRI with MR angiography was performed in VM patients 3–6 days after clinical exam to rule out brainstem, cerebellar lesions, or vertebrobasilar TIA when their clinical exam was suggestive of a central lesion (i.e. video-oculographic confirmed gaze-evoked nystagmus).

A board-certificated neurologist performed a structured clinical examination on each patient to include: 1) Subjective Visual Vertical (SVV) using the bucket test, 2) ABCD^2^, 3) headache and vertigo history, 4) focal neurological (i.e. proprioception, vision) exam, 5) video-oculography examining for spontaneous, gaze evoked, positional, and head shaking-induced nystagmus, and 6) clinical head impulse testing. Healthy control subjects completed only the SVV test.

### Subjective visual vertical using bucket test

Deviation of the SVV is a clinical sign of a deficit involving the graviceptive pathways [23]. Tests of SVV are widely used in neuro-otological examinations to detect the dysfunction of otolith organs and vertical semicircular canals, and to help diagnose central vestibular disorders [24–26]. The test is performed with subjects seated upright looking into an opaque plastic bucket, with the head placed inside the rim of the bucket to prevent visual orientation cues. A straight, yellow diametric line is placed on the interior and bottom of the bucket. On the exterior, the bottom of the bucket held a protractor (180°), with a zero line at 90° corresponding to the true vertical. A weighted string was suspended from the center of the bucket bottom and served as the plumb line for which the reading was made [27]. For each measurement of SVV, the examiner rotated the bucket to an initial displacement, and from there the subject rotated the bucket clockwise or counterclockwise to an endpoint, stopping when the inside line appeared to be vertical (Additional file 1: Figure S1). The examiner noted the position of the plumb line on the protractor. Three trials were performed, with an inter-trial interval of 1 minute. Mean values of the SVV deviations were calculated for all subjects. We defined the normal range of SVV deviation as determined by the bucket test to be 0 ± 2.3° based on the literature [27].

### ABCD^2^

The ABCD^2^ exam (Additional file 3: Table S2) combines points for Age, Blood pressure, Clinical features, Duration of symptoms, and presence of Diabetes as means to help predict the risk of stroke after having a transient ischemic attack. ABCD^2^ scores ≧4 are defined as having a higher risk of a future cerebrovascular event [28].

### Nystagmus

Spontaneous and gaze-evoked nystagmus (i.e. the nystagmus induced by eccentric gaze 30 degrees from central position) in upright and seated position was first examined in room light. Next, video-oculography (Synapsys, France) was employed and these two exams were repeated, along with Dix-Hallpike test, supine roll test, and the head-shaking nystagmus test.

### Focal neurological signs

Formal neurological examinations were performed on all patients, including clinical assessments for cognition and cranial nerves, manual muscle tests, deep tendon reflex and Babinski’s sign, sensory tests (pinprick, light touch, vibration, and joint position sense), tests for limb ataxia (finger-to-nose test, heel-to-shin test, finger tapping, and foot tapping), and gait. The definition of having abnormal focal neurological signs was the presence of one or more of the following, (i) dysfunction of cranial nerves, (ii) weakness or upper motor neuron signs, (iii) sensory defects, or (iv) limb ataxia.

Postural imbalance or unsteadiness, which may appear in the acute stage of various vestibular disorders, were not included as focal neurological signs. Alteration of consciousness or other cognitive impairments were also excluded as a positive focal neurological sign, as they were more likely to be affected by diffuse cortical or non-neurological processes such as metabolic disturbances or drug effects.

### Head impulse test

Horizontal head impulse testing was performed at the bedside. Patients were instructed to look at the examiner’s nose, and the examiner quickly turned the patient’s head with small amplitude, moderate velocity, and high acceleration head rotations while observing the patients’ eyes. The existence of a re-fixation saccade was defined as “positive.” Repetitive head impulses of unpredictable timing and direction were applied in attempt to reduce the presence of covert saccades and increase the test sensitivity [29].

### Statistical analysis

All data was assessed for normality. Student’s t-test was used to examine continuous data between groups. For non-parametric data, we used the Mann-Whitney U-test (i.e., absolute deviation of SVV). Chi-squared analysis and the Fisher’s exact test compared the categorical variables between the groups. In order to determine how well the SVV bucket test distinguished CVD from VM, we used the receiver-operating characteristic (ROC) curve and calculated the area under the ROC curve (AUC). For investigation of diagnostic accuracy, the sensitivity, specificity, positive likelihood ratio and negative likelihood ratio of our different clinical measures were determined and compared using the McNemar test. The comparisons were set at a minimum significance level (α) to 0.05, but for multiple comparisons between CVD and VM we performed a Bonferroni correction with an α-level adjusted to a minimum of 0.0041. Statistical significance was assessed with SPSS (version 23) (IBM SPSS Inc., Chicago, IL, USA).

## Results

### Demographic data, ABCD^2^, and headache

The demographic data and clinical characteristics of enrolled patients are listed in Table 1. When compared with the VM group, patients in the CVD group had 1) significantly higher age, 2) lower female/male ratio, 3) higher proportion of diabetes, 4) higher proportion of hypertension, and 5) fewer headaches preceding or following the attack of vestibular symptoms. The ABCD^2^ was significantly higher in the CVD group compared with the VM group (*p* < 0.001). 51.9% of CVD patients (54.1% excluding nonvascular etiologies) in contrast to 5.6% of VM patients had ABCD^2^ ≧4. Although accompanying headache is one of the characteristics of VM, only 58.3% of VM patients complained of headache preceding or following the attack of vestibular symptoms.

**Table 1:** Demographic and clinical test data in CVD and VM

	CVD (*n* = 27)	VM (*n* = 36)	*p* value
Age, mean years ±1SD	57.0 ± 16.7	43.0 ± 15.8	< 0.001
Female, n (%)	8 (29.6%)	31 (86.1%)	< 0.001
Diabetes, n (%)	10 (37.0%)	2 (5.6%)	0.002
Hypertension, n (%)	17 (63.0%)	3 (8.3%)	< 0.001
ABCD2≧4, n (%)	14 (51.9%)	2 (5.6%)	< 0.001
Headache^a^, n (%)	4 (14.8%)	21 (58.3%)	< 0.001
Absolute SVV, mean ± 1SD	4.8 ± 4.1°	0.7 ± 1.0°	< 0.001
Patients with SVV > 2.3°, n (%)	20 (74.1%)	3 (8.3%)	< 0.001
Focal neurological signs, n (%)	11 (40.7%)	1 (2.8%)	< 0.001
Nystagmus in room light, n (%)	9 (33.3%)	0 (0)	–
Nystagmus with fixation blocked, n (%)	13 (48.1%)	16 (44.4%)	0.77
Head impulse test (%)	3 (11.1%)	0 (0)	–

*CVD* central vestibular disorders, *VM* vestibular migraine, *SVV* subjective visual vertical

^a^Headache preceded or followed the vestibular attack

Etiologies in the CVD group included infarction (*n* = 22), hemorrhage (*n* = 2), and multiple sclerosis (*n* = 3). Nineteen of 27 participants were male. Lesions were localized to the medulla oblongata (*n* = 4), pons (*n* = 7), cerebellum (*n* = 11), or multiple regions (*n* = 5). The duration of symptom onset and clinical examination was within 3 days (*n* = 7), 4–10 days (*n* = 9), or more than 10 days (*n* = 11) (Table 2).

**Table 2:** Clinical features of the patients with CVD

Age Group^a^	From onset to assessment	Diagnosis	Location	Symptoms/signs	Nystagmus in room light	Nystagmus with fixation blocked	SVV	Head impulse test
E	3 days	Infarction	Right medulla	Isolated vertigo	None	None	15.7°, R	Negative
B	4 days	Infarction	Multiple areas	Isolated vertigo	GEN	GEN	15.7°, R	Positive, L
A	2 weeks	Infarction	Left medulla	Vertigo, facial numbness	GEN	GEN	11.0°, L	Positive, L
D	3 days	Infarction	Right pons	Vertigo, dysmetria	Right-beating SN	Right-beating SN	9.0°, L	Negative
B	3 days	Infarction	Bilateral cerebellum	Isolated vertigo	Right-beating SN	Right-beating SN	8.0°, L	Negative
D	2 weeks	Infarction	Left medulla	Vertigo, hemiparesthesia	None	Right-beating SN	6.7°, L	Negative
D	3 days	Infarction	Right cerebellum	Isolated vertigo	None	Right-beating HSN	6.0°, L	Negative
A	3 weeks	Infarction	Right cerebellum	Isolated vertigo	None	None	5.0°, R	Negative
E	7 days	Infarction	Right cerebellum	Isolated vertigo	None	None	5.0°, L	Negative
A	10 days	Infarction	Left pons	Vertigo, dysarthria	None	None	4.7°, L	Negative
E	8 days	Infarction	Left pons	Vertigo, hemiparesis	None	None	4.7°, L	Negative
E	3 weeks	Infarction	Bilateral pons	Vertigo, hemiparesis	None	None	4.3°, R	Negative
B	3 days	Infarction	Right cerebellum	Isolated vertigo	Right-beating SN	Right-beating SN	4.3°, L	Negative
C	3 weeks	Infarction	Multiple areas	Isolated vertigo	None	None	4.3°, R	Negative
B	2 weeks	Multiple sclerosis	Left medulla	Isolated vertigo	Downbeat SN	Downbeat SN	3.7°, L	Negative
B	7 days	Infarction	Right cerebellum	Isolated vertigo	None	None	3.7°, L	Negative
B	1 month	Infarction	Bilateral cerebellum	Isolated vertigo	GEN	Left-beating SN, GEN	3.0°, L	Positive, L
D	6 days	Hemorrhage	Left cerebellum	Vertigo, dysmetria	None	Left-beating HSN	3.0°, R	Negative
E	2 weeks	Infarction	Right cerebellum	Isolated vertigo	None	None	2.7°, L	Negative
C	3 weeks	Infarction	Left pons	Isolated vertigo	None	None	2.3°, L	Negative
D	4 days	Infarction	Bilateral pons	Isolated vertigo	None	None	2.0°, L	Negative
D	8 days	Multiple sclerosis	Multiple areas	Vertigo, hemianopia	None	None	1.7°, R	Negative
C	2 weeks	Multiple sclerosis	Multiple areas	Vertigo, dysarthria	GEN	Left-beating HSN, PN	1.3°, L	Negative
C	2 days	Infarction	Multiple areas	Isolated vertigo	None	None	1.0°, L	Negative
C	1 month	Infarction	Left cerebellum	Isolated vertigo	Left-beating SN	Left-beating SN, HSN	0.3°, R	Negative
D	2 days	Infarction	Left pons	Vertigo, facial palsy, dysarthria	None	None	0°	Negative
E	4 days	Hemorrhage	Left cerebellum	Vertigo, dysmetria	None	Left-beating HSN	0°	Negative

*M* male, *F* female, *B* bilateral, *GEN* gaze-evoked nystagmus, *SN* spontaneous nystagmus, *HSN* head-shaking nystagmus, *PN* positional nystagmus, *SVV* subjective visual vertical

^a^Age grouping: A, 30–41 years; B, 42–51 years; C, 51–60 years; D, 61–71 years; E, > 72 years

In the VM group, 21 patients were diagnosed with “definite” and 15 patients were diagnosed with probable VM, per the criteria specified in the ICVD [7]. The duration of symptom onset and clinical examination was between 2 h and 2 days. Nine patients were found to have central ocular motor signs during video-oculography, including weak downbeat nystagmus (*n* = 2), perverted head shaking nystagmus (*n* = 2), and central positional nystagmus (*n* = 5) (Additional file 4: Table S3). They underwent MRI with MR angiography; all showed normal results.

### Subjective visual vertical

**Fig. 1:**
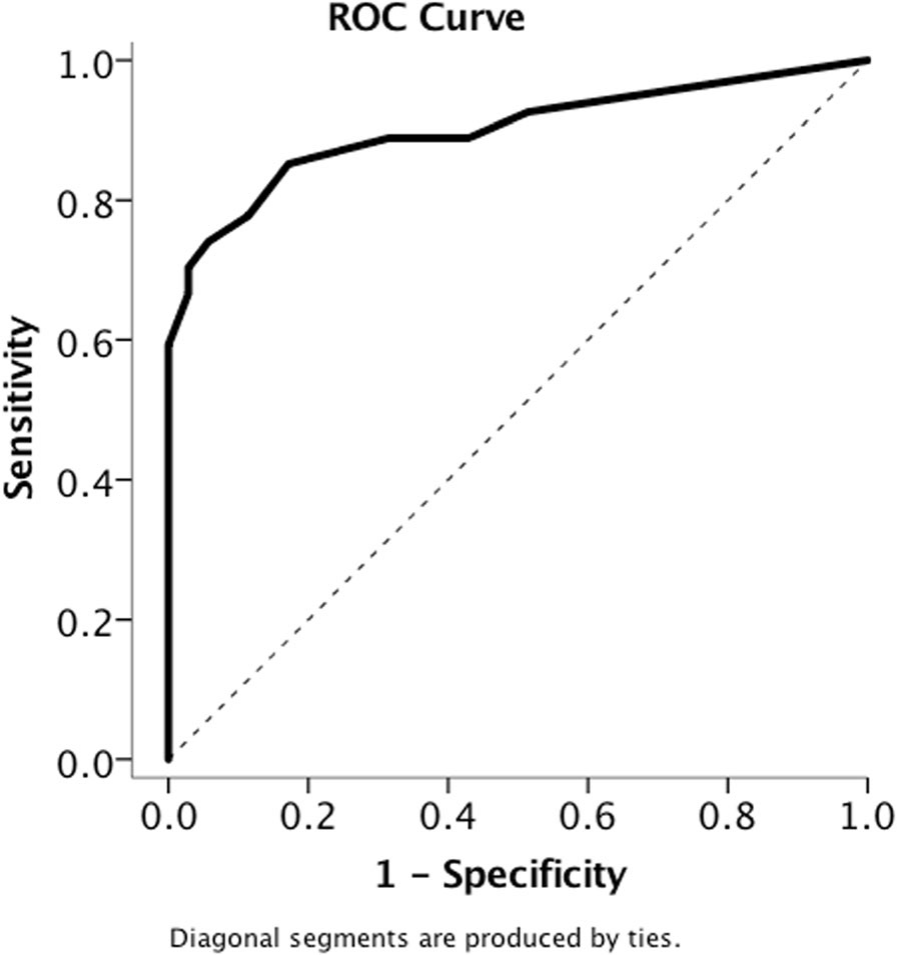
ROC curve of SVV for differentiating CVD from VM. The area under the curve was 0.9, and the best threshold was 2.2. (solid line – ROC curve; dotted line - reference line)

The absolute deviation of the SVV in the CVD group was 4.8 ± 4.1°, much larger than the SVV deviation in age/gender-matched healthy controls (0.9 ± 1.0°; Mann-Whitney U-test; *p* < 0.001) (SVV results for healthy controls are listed in Additional file 5: Table S4). The absolute deviation of SVV in the VM group was 0.7 ± 1.0°, much smaller than the SVV deviations in the CVD group (Mann-Whitney U-test; *p* < 0.001) (Table 1). ROC analysis revealed an AUC of 0.9, demonstrating excellent discrimination for SVV to differentiate CVD from VM. The best threshold to detect SVV deviations outside the normal range was 2.2°, a value nearly equivalent to those cited as the normal range of SVV deviation (±2.3°) for healthy subjects in the literature [27] (Fig. 1).

With the normal range of SVV deviation for the bucket test set at ±2.3° from the vertical [27], the abnormal rate of SVV deviation in the CVD group was found to be significantly higher than that of the VM group (74.1% VS 8.3%; Chi-Square test; *p* < 0.001) (Table 1).

Despite heterogeneous etiologies in the CVD group, 24 of the 27 patients who were diagnosed with stroke (22 with infarction and 2 with hemorrhage) had a higher abnormal rate of SVV deviation than the VM group (75% vs 8.3%; Chi-Square test; *p* < 0.001). Although the intervals between onset and assessment were different in the CVD group, 7 patients who were assessed within 3 days after onset had mean SVV deviation 6.3°, 9 who were assessed 4–10 days after onset had mean SVV deviation 4.5°, and 11 patients who were assessed more than 10 days after onset had mean SVV deviation 4.1°. In the CVD subgroups assessed at different stages (≤ 3 days, 4–10 days, and > 10 days), the abnormal rates of SVV deviation were all higher than that of the VM group (Fisher’s exact test; *p* < 0.005). Seven patients in CVD group had significant deviation of SVV, which was the only abnormal findings in their clinical tests (Table 2).

### Other clinical tests

Of the 27 patients diagnosed with CVD, 11 patients (40.1%) presented with focal neurological signs. By contrast, only one patient with VM (2.8%) presented with a focal neurological sign (visual field defect) preceding the onset of vertigo. When nystagmus was observed in room light (unaided), nystagmus was present in 9 of 27 CVD patients (33.3%) and included unidirectional horizontal nystagmus (*n* = 4), gaze-evoked nystagmus (*n* = 4), and vertical nystagmus (*n* = 1). Unlike the CVD group, the room light exam for nystagmus in the patients with VM was normal. However, during video-oculography (fixation blocked), 16 of the 36 VM patients (44.4%) showed a weak spontaneous nystagmus (*n* = 4); persistent positional nystagmus atypical for benign paroxysmal positional vertigo (*n* = 10), and/or head-shaking induced nystagmus (*n* = 8) (Additional file 4: Table S3). Nystagmus with fixation blocked was not able to differentiate CVD from VM (Chi-Square test; *p* = 0.77). Clinical head impulse testing was abnormal (presence of corrective saccades) in only three (11.1%) of the CVD patients, and none of patients with VM (Table 1). The three CVD patients with abnormal HIT had left lateral pontine/right cerebellar infarcts, left lateral medullary infarct, and bilateral cerebellar infarcts respectively.

### Diagnostic accuracy

The sensitivity, specificity, positive likelihood ratio and negative likelihood ratio of the bucket test alone was 74.1, 91.7%, 8.9, and 0.3 respectively. Compared with other diagnostic tests, SVV was the most sensitive (McNemar test; *p* < 0.05), but not the most specific (Table 3).

**Table 3:** Diagnostic accuracy for distinguishing CVD from VM

Diagnostic tool	Sensitivity, % (95% CI)	Specificity, % (95% CI)	Positive likelihood ratio (95% CI)	Negative likelihood ratio (95% CI)
Clinical examinations				
Abnormal SVV deviation^a^	74.1% (55.3–86.8%)	91.7% (78.2–97.1%)	8.9 (2.9–26.9)	0.3 (0.1–0.5)
Focal neurological signs	40.7% (24.5–59.3%)	97.2% (85.8–99.5%)	14.7 (2.0–106.8)	0.6 (0.4–0.8)
Nystagmus in room light	33.3% (18.6–52.2%)	100% (90.4–100%)	Undefined^b^	0.7 (0.5–0.9)
Combination of three signs^c^	92.6% (76.6–97.9%)	88.9% (74.7–95.6%)	8.3 (3.3–21.1)	0.08 (0.02–0.3)
Head impulse test	11.1% (3.9–28.1%)	100% (90.4–100%)	Undefined	0.9 (0.8–1.0)
History				
ABCD2	51.9% (34.0–69.3%)	94.4% (81.9–98.5%)	9.3 (2.3–37.7)	0.5 (0.3–0.8)
No headache	85.2% (67.5–94.1%)	58.3% (42.2–72.9%)	2 (1.3–3.1)	0.3 (0.1–0.7)
Combination of all the histories/tests above	100% (87.5–100%)	50% (34.5–65.5%)	2 (1.4–2.8)	0

^a^Abnormal SVV deviation means absolute deviation of SVV greater than 2.3°

^b^When specificity is 100%, positive likelihood ratio is undefined

^c^Presence of abnormal SVV deviation, focal neurological signs, or nystagmus during room light exam provide the optimal diagnostic accuracy

In order to improve diagnostic accuracy, we combined SVV with the other clinical tests to help differentiate CVD from VM. When we combined the presence of an abnormal SVV deviation as measured by the bucket test, abnormal focal neurological signs, or nystagmus observed in room light (the presence of all three was defined as positive while the absence of all three was defined as “negative”), sensitivity improved to 92.6%, while the specificity remained relatively stable (88.9%). The inclusion of head impulse, headache history, and the ABCD^2^ did not improve accuracy (Table 3).

## Discussion

In this study, we consider the unique and combined benefit of standard bedside ocular motor examinations and medical histories to distinguish CVD from VM. Our study found that the SVV bucket test was the single, most sensitive bedside exam for identifying CVD in these difficult patients. However, the addition of a positive neurologic exam and gaze instability optimized our ability to distinguish an elusive CVD from VM.

Abnormal SVV is proposed as a sensitive sign of brainstem pathology [23], but is also abnormal in patients with cerebellar lesions [30]. Unfortunately, patients with acute peripheral vestibular hypofunction also can have an abnormal perception of vertical and thus SVV is considered insensitive to distinguish central from peripheral causes of vestibular disorders [31]. Early studies of SVV in patients with migraine have either combined VM with peripheral etiologies, only included patients with migraine headache but not vertigo, or measured SVV during the interictal period [32–34]. Those reports showed no significant deviations, or only very subtle, non-pathological deviations. Two recent studies have shown that patients with vestibular migraine have SVV similar with normal controls, as we report [35, 36]. Similar with prior data, rarely did we find that VM can cause an abnormal SVV deviation (2/36; 5.6%) during an acute episode. The reason for why patients with CVD have abnormal SVV but those with VM do not, is unknown but may related to structural versus functional differences in the lesion and/or the involvement of unilateral or bilateral vestibular pathways.

For our study groups, sensitivity (74.1%) and specificity (91.7%) of the SVV bucket test alone was roughly comparable to the findings from the nystagmus and focal neurological examinations alone. However, the bucket test as performed in our study is not accurate enough to be an independent diagnostic tool, given its sensitivity was not high enough to identify CVD.

**Fig. 2:**
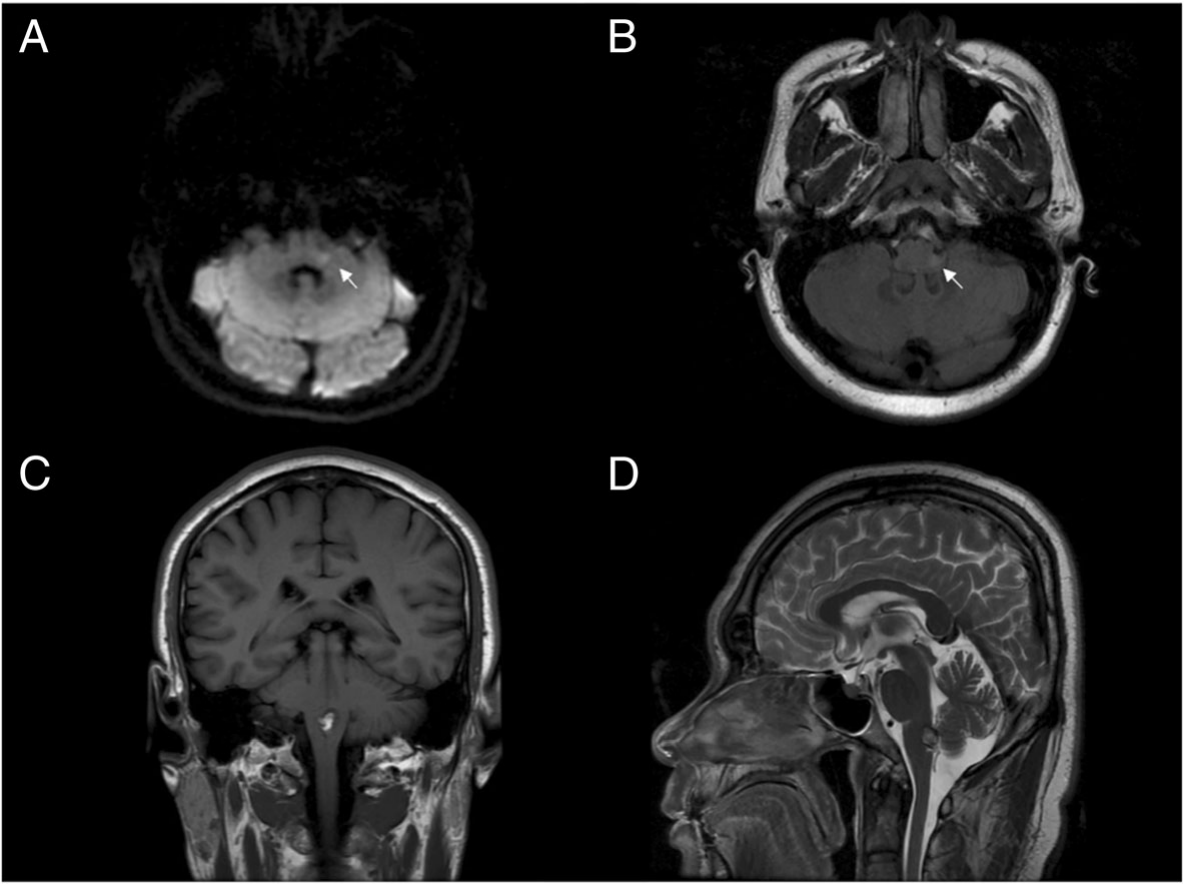
Two cases of unremarkable focal neurological or nystagmus findings but marked SVV deviations. **a**, **b** A young man (30–35 years) complaining of dizziness only had equivocal facial numbness and end-gaze nystagmus. However, the bucket test revealed an SVV deviation of 11 degrees to the left; MRI showed a tiny infarct in the left lateral medulla (arrow). **c**, **d** An older man (50–55 years) with 10 days of dizziness but no focal neurological signs or nystagmus: His bucket test showed an SVV deviation of 5 degrees to the left; MRI showed a cavernous hemangioma in the dorsal medulla

Traditionally, CVD has been identified by the presence of focal neurological signs. However, this concept has been challenged in recent years following the publication of several reports demonstrating that small lesions in the nodulus, uvula, lateral medulla or dorsal pons can create vertigo in the absence of neurological signs [18–21]. In a large prospective study (*n* = 101), only 19% of CVD patients had focal neurological signs (e.g. 51% with truncal ataxia) [1]. Similarly, in our study focal neurological signs were observed in only 40.7% of the CVD patients, much less than those showing an abnormal SVV (74.1%). The fact that some of the CVD patients failed to exhibit apparent neurological signs or nystagmus but had a marked deviation of SVV suggests a covert and potentially dangerous imbalance of the graviceptive pathways (see the examples in Fig. 2). While there is no precedent for replacing the standard neurological examination, combining the SVV bucket test with results from a nystagmus exam and the focal neurological exam significantly sharpens the diagnostic accuracy to distinguish CVD from VM. It remains possible that focal neurological signs can betray the auras of basilar-type or hemiplegic migraine. But these migraine variants are supposed to be rare, or at least their prevalence is substantially lower than that of VM.

Nystagmus is an important sign for diagnosing vestibular disorders, and in our study, 33.3% of CVD patients presented with nystagmus when examined directly in room light. By contrast, none of the 36 VM patients had nystagmus appreciable in room light (unaided), though 16 patients did have weak nystagmus on video-oculography (fixation blocked). These findings are compatible with the study of Polensak and Tusa, in which all of the 26 patients with acute VM failed to exhibit spontaneous or gaze-evoked nystagmus without removal of visual fixation [11]. Although VM patients show certain features of central-type ocular motor dysfunction that can be indistinguishable from CVD [9], in our study the nystagmus associated with VM was suppressed by visual fixation, which is atypical in patients with CVD [37, 38].

Compatible with prior studies, we found that the vascular risk score ABCD^2^ was not sensitive for detecting CVD [28]. We also show that nearly a half of patients with VM did not present with headache within close proximity of their vestibular symptoms [6]. These results suggest that differentiation between CVD and VM is not possible by history alone. In addition, we found that head impulse testing alone (sensitivity 11.1%), which can be useful in differentiating central from peripheral causes of vestibular symptoms, was not useful for differentiating CVD from VM.

### Limitations

There exist a few limitations associated with this study. Vertigo due to an asymmetry in the resting firing rates of each vestibular system is a dynamic process and its associated signs may have changed by the time the exams were conducted. This may have led to an underestimation of either the abnormal SVV deviations, focal neurological signs, or nystagmus. This is unlikely to have had a significant impact given that each of the patients consented in our study were symptomatic during clinical assessment. Second, the duration between vertigo onset and examination was different between the CVD and VM groups. Despite the longer duration, patients in the CVD group still had higher rates of abnormal SVV deviations, abnormal nystagmus and positive focal neurological signs compared with the VM group. It remains possible though, that if the patients with CVD were examined closer to the time from onset, the magnitude of their abnormal examinations would have been greater. Third, recent literature suggests 6–10 repetitions of SVV testing, evenly applied to both sides is optimal [39, 40]. Thus it is possible that were we to have used a greater number of SVV repetitions, diagnostic accuracy of SVV alone in differentiating CVD from VM may have improved. Finally, while adequate, the sample size is not very large and thus we cannot exclude the existence of sampling bias. Larger, community-based studies are warranted to confirm and extend our results to the general population.

## Conclusion

This study found the combined clinical findings of perception of a tilted vertical (SVV bucket test > 2.3°), a positive neurological exam, and gaze stability being abnormal (spontaneous or gaze-evoked nystagmus with visual fixation) has high sensitivity and specificity to distinguish CVD from VM. The addition of the head impulse test, the ABCD2, and history of headache may be helpful to inform the diagnosis but do not improve diagnostic efficiency. In the case of acute vertigo presentations, the HINTS examination remains the most important tool for distinguishing central from peripheral causes. However, in the case of a normal head impulse test, a pathophysiological reason such as VM may be the cause – in which case the diagnostic battery combining bucket test, focal neurological signs and nystagmus is very useful.

## Additional files

Additional file 1: **Figure S1.** Bucket test for examining subjective visual vertical [27]. (DOCX 969 kb)

Additional file 2: **Table S1.** Diagnostic criteria of vestibular migraine [7]. (DOCX 13 kb)

Additional file 3: **Table S2.** ABCD2 scoring system [28]. (DOCX 13 kb)

Additional file 4: **Table S3.** Clinical features of the patients with VM. (DOCX 24 kb)

Additional file 5: **Table S4.** The results of subjective visual vertical for healthy controls matching the age and sex in CVD. (DOCX 15 kb)

## Data Availability

The datasets are available from the corresponding author on reasonable request.

## Abbreviations

AUC: Area under curve
CVD: Central vestibular disorder
HINTS: Head Impulse test, Nystagmus, and Test of Skew deviation
ICVD: International Classification of Vestibular Disorders
IHS: International Headache Society
ROC: Receiver operating characteristic
SVV: Subjective visual vertical
TIA: Transient ischemic attack
VM: Vestibular migraine

## References

[1] Kattah JC, Talkad AV, Wang DZ, Hsieh Y-H, Newman-Toker DE (2009). HINTS to diagnose stroke in the acute vestibular syndrome: three-step bedside oculomotor examination more sensitive than early MRI diffusion-weighted imaging. Stroke.

[2] Carmona S, Martínez C, Zalazar G, Moro M, Batuecas-Caletrio A, Luis L, et al. The diagnostic accuracy of truncal Ataxia and HINTS as cardinal signs for acute vestibular syndrome. Front Neurol. 2016;7. 10.3389/fneur.2016.00125. 10.3389/fneur.2016.00125 PMC4976483 27551274

[3] Vanni S, Pecci R, Casati C, Moroni F, Risso M, Ottaviani M (2014). STANDING, a four-step bedside algorithm for differential diagnosis of acute vertigo in the emergency department. Acta Otorhinolaryngol Ital.

[4] Neuhauser H, Leopold M, Von Brevern M, Arnold G, Lempert T (2001). The interrelations of migraine, vertigo, and migrainous vertigo. Neurology.

[5] Lardreau E (2012). A curiosity in the history of sciences: the words “megrim” and “migraine”. J Hist Neurosci.

[6] Neuhauser H, Lempert T (2004). Vertigo and dizziness related to migraine: a diagnostic challenge. Cephalalgia.

[7] Lempert T, Olesen J, Furman J, Waterston J, Seemungal B, Carey J (2012). Vestibular migraine: diagnostic criteria. J Vestib Res.

[8] Blödow A, Heinze M, Bloching MB, von Brevern M, Radtke A, Lempert T (2014). Caloric stimulation and video-head impulse testing in Ménière’s disease and vestibular migraine. Acta Otolaryngol.

[9] von Brevern M, Zeise D, Neuhauser H, Clarke AH, Lempert T. Acute migrainous vertigo: clinical and oculographic findings. Brain 2005;128 Pt 2:365–374. 10.1093/brain/awh351 15601663

[10] Power L, Shute W, McOwan B, Murray K, Szmulewicz D (2018). Clinical characteristics and treatment choice in vestibular migraine. J Clin Neurosci.

[11] Polensek SH, Tusa RJ (2010). Nystagmus during attacks of vestibular migraine: an aid in diagnosis. Audiol Neuro Otol.

[12] Sturzenegger M (1994). Headache and neck pain: the warning symptoms of vertebral artery dissection. Headache.

[13] Grad A, Baloh RW (1989). Vertigo of vascular origin: clinical and Electronystagmographic features in 84 cases. Arch Neurol.

[14] Olesen J (2005). Vertigo and dizziness related to migraine: a diagnostic challenge. Cephalalgia.

[15] Li L, Schulz UG, Kuker W, Rothwell PM, Oxford Vascular Study (2015). Age-specific association of migraine with cryptogenic TIA and stroke: population-based study. Neurology.

[16] Waterston J (2004). Chronic migrainous vertigo. J Clin Neurosci.

[17] Carvalho GF, Vianna-Bell FH, Florencio LL, Pinheiro CF, Dach F, Bigal ME (2018). Presence of vestibular symptoms and related disability in migraine with and without aura and chronic migraine. Cephalalgia.

[18] Kim JS (2000). Vertigo and gait ataxia without usual signs of lateral medullary infarction: a clinical variant related to rostral-dorsolateral lesions. Cerebrovasc Dis.

[19] Lee H, Sohn S-I, Cho Y-W, Lee S-R, Ahn B-H, Park B-R (2006). Cerebellar infarction presenting isolated vertigo: frequency and vascular topographical patterns. Neurology.

[20] Moon IS, Kim JS, Choi KD, Kim M-J, Oh S-Y, Lee H (2009). Isolated nodular infarction. Stroke.

[21] Chang T-P, Wu Y-C (2010). A tiny infarct on the dorsolateral pons mimicking vestibular neuritis. Laryngoscope.

[22] Kerber KA, Newman-Toker DE (2015). Misdiagnosing dizzy patients: common pitfalls in clinical practice. Neurol Clin.

[23] Dieterich M, Brandt T (1993). Ocular torsion and tilt of subjective visual vertical are sensitive brainstem signs. Ann Neurol.

[24] Pinar HS, Ardiç FN, Topuz B, Kara CO (2005). Subjective visual vertical and subjective visual horizontal measures in patients with chronic dizziness. J Otolaryngol.

[25] Piscicelli C, Pérennou D (2017). Visual verticality perception after stroke: a systematic review of methodological approaches and suggestions for standardization. Ann Phys Rehabil Med.

[26] Dieterich M, Brandt T. Perception of verticality and vestibular disorders of balance and falls. Front Neurol. 2019;10. 10.3389/fneur.2019.00172. 10.3389/fneur.2019.00172 PMC6457206 31001184

[27] Zwergal A, Rettinger N, Frenzel C, Dieterich M, Brandt T, Strupp M (2009). A bucket of static vestibular function. Neurology.

[28] Navi BB, Kamel H, Shah MP, Grossman AW, Wong C, Poisson SN (2012). Application of the ABCD2 score to identify cerebrovascular causes of dizziness in the emergency department. Stroke.

[29] Tjernström F, Nyström A, Magnusson M (2012). How to uncover the covert saccade during the head impulse test. Otol Neurotol.

[30] Baier B, Bense S, Dieterich M (2008). Are signs of ocular tilt reaction in patients with cerebellar lesions mediated by the dentate nucleus?. Brain.

[31] Min KK, Ha JS, Kim MJ, Cho CH, Cha HE, Lee JH (2007). Clinical use of subjective visual horizontal and vertical in patients of unilateral vestibular neuritis. Otol Neurotol.

[32] Kandemir A, Çelebisoy N, Köse T (2014). Perception of verticality in patients with primary headache disorders. J Int Adv Otol.

[33] Crevits L, Vanacker L, Verraes A (2012). Patients with migraine correctly estimate the visual verticality. Clin Neurol Neurosurg.

[34] Asai M, Aoki M, Hayashi H, Yamada N, Mizuta K, Ito Y (2009). Subclinical deviation of the subjective visual vertical in patients affected by a primary headache. Acta Otolaryngol.

[35] Miller MA, Crane BT (2016). Static and dynamic visual vertical perception in subjects with migraine and vestibular migraine. World J Otorhinolaryngol Head Neck Surg.

[36] Ashish G, Augustine AM, Tyagi AK, Lepcha A, Balraj A (2017). Subjective visual vertical and horizontal in vestibular migraine. J Int Adv Otol.

[37] Takemori S, Cohen B (1974). Loss of visual suppression of vestibular nystagmus after flocculus lesions. Brain Res.

[38] Kim H-A, Yi H-A, Lee H (2016). Failure of fixation suppression of spontaneous nystagmus in cerebellar infarction: frequency, pattern, and a possible structure. Cerebellum.

[39] Pérennou D, Piscicelli C, Barbieri G, Jaeger M, Marquer A, Barra J (2014). Measuring verticality perception after stroke: why and how?. Neurophysiol Clin.

[40] Piscicelli C, Nadeau S, Barra J, Pérennou D (2015). Assessing the visual vertical: how many trials are required?. BMC Neurol.

