# Supplementary material for: The bucket test differentiates patients with MRI confirmed brainstem/cerebellar lesions from patients having migraine and dizziness alone
Source: BMC Neurol. 2019 Sep 3;19:219. doi: 10.1186/s12883-019-1442-z (PMC6720090; doi:10.1186/s12883-019-1442-z)
Supplement: Supplementary file 1 — Figure S1. Bucket test for examining subjective visual vertical [27]. (DOCX 969 kb) [file 12883_2019_1442_MOESM1_ESM.docx]

**Additional Fig. 1.** Bucket test for examining subjective visual vertical


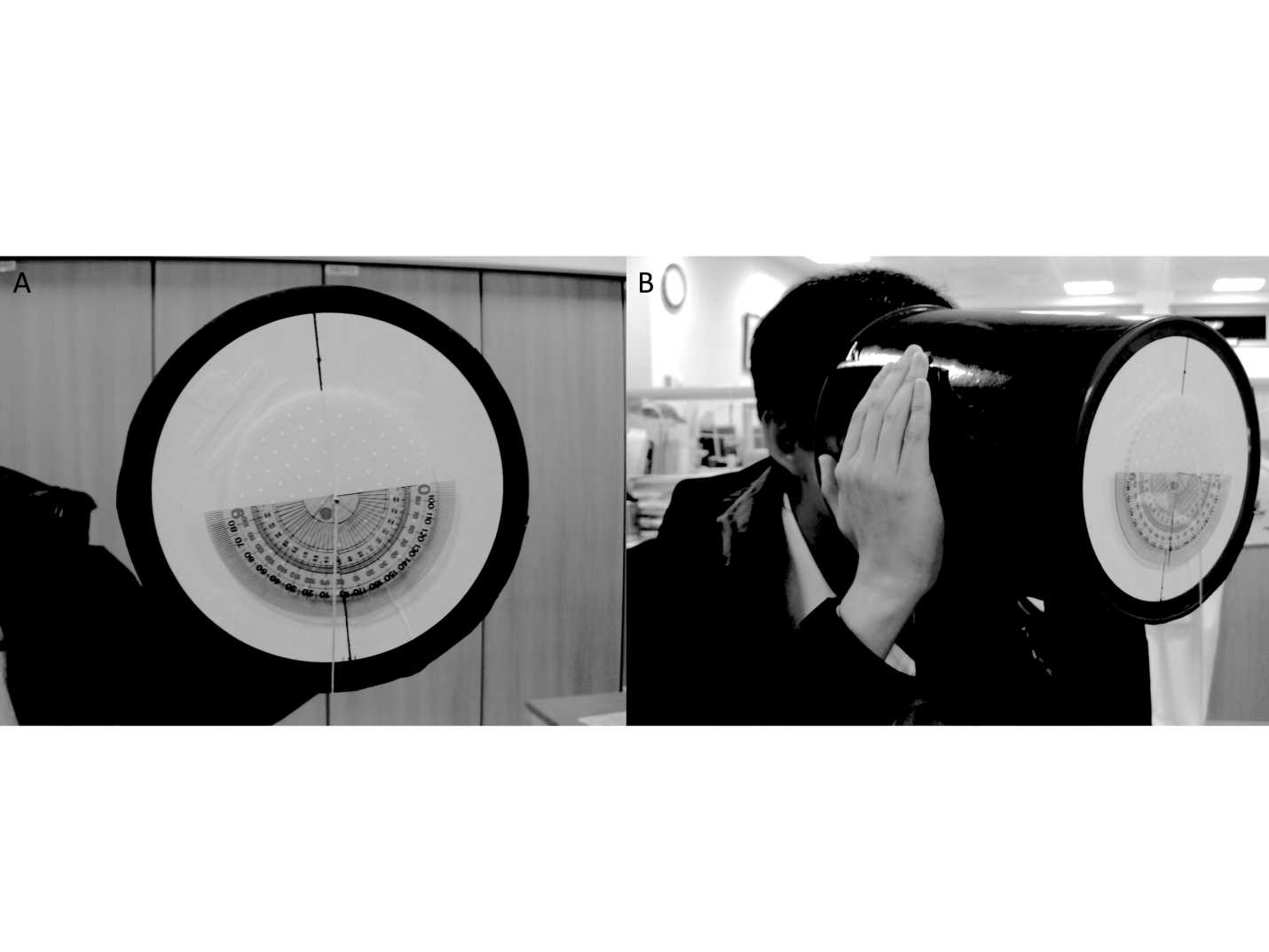


**(A)** A perpendicular line orignates from the center of the bucket bottom. This line and the zero line form an angle which represents the deviation of visual vertical. **(B)** Subjects rotate the bucket clockwise or counterclockwise to an end position where they estimate the inside bottom line to be truly vertical.
