# Supplementary material for: The bucket test differentiates patients with MRI confirmed brainstem/cerebellar lesions from patients having migraine and dizziness alone
Source: BMC Neurol. 2019 Sep 3;19:219. doi: 10.1186/s12883-019-1442-z (PMC6720090; doi:10.1186/s12883-019-1442-z)
Supplement: Supplementary file 2 — Table S1. Diagnostic criteria of vestibular migraine [7]. (DOCX 13 kb) [file 12883_2019_1442_MOESM2_ESM.docx]

**Additional Table 1**. Diagnostic criteria of vestibular migraine

| *1. Vestibular migraine* | |
| --- | --- |
| A. At least 5 episodes with vestibular symptoms of moderate or severe intensity, lasting 5 min to 72 hours | |
| B. Current or previous history of migraine with or without aura according to the International Classification of Headache Disorders (ICHD) | |
| C. One or more migraine features with at least 50% of the vestibular episodes: | |
| **–** headache with at least two of the following characteristics: one sided location, pulsating quality, moderate or severe pain intensity, aggravation by routine physical activity |  |
| **–** photophobia and phonophobia |  |
| **–** visual aura |  |
| D. Not better accounted for by another vestibular or ICHD diagnosis | |
| *2. Probable vestibular migraine* | |
| A. At least 5 episodes with vestibular symptoms of moderate or severe intensity, lasting 5 min to 72 hours | |
| B. Only one of the criteria B and C for vestibular migraine is fulfilled (migraine history *or* migraine features during the episode) | |
| C. Not better accounted for by another vestibular or ICHD diagnosis | |
