# Supplementary material for: The bucket test differentiates patients with MRI confirmed brainstem/cerebellar lesions from patients having migraine and dizziness alone
Source: BMC Neurol. 2019 Sep 3;19:219. doi: 10.1186/s12883-019-1442-z (PMC6720090; doi:10.1186/s12883-019-1442-z)
Supplement: Supplementary file 3 — Table S2. ABCD2 scoring system [28]. (DOCX 13 kb) [file 12883_2019_1442_MOESM3_ESM.docx]

**Additional Table 2.** ABCD^2^ scoring system

| Risk factor | Points |
| --- | --- |
| Age≧60 | 1 |
| Blood pressure: Systolic≧140 or diastolic≧90 | 1 |
| Clinical features: Unilateral weakness | 2 |
| Speech disturbance | 1 |
| Duration: ≧60 minutes | 2 |
| 1-59 minutes | 1 |
| Diabetes mellitus | 1 |
