# Supplementary material for: The bucket test differentiates patients with MRI confirmed brainstem/cerebellar lesions from patients having migraine and dizziness alone
Source: BMC Neurol. 2019 Sep 3;19:219. doi: 10.1186/s12883-019-1442-z (PMC6720090; doi:10.1186/s12883-019-1442-z)
Supplement: Supplementary file 4 — Table S3. Clinical features of the patients with VM. (DOCX 24 kb) [file 12883_2019_1442_MOESM4_ESM.docx]

**Additional Table 3.** Clinical features of the patients with VM

| **Age Group** | **From onset to assessment** | **Diagnosis** | **Symptoms/signs** | **Nystagmus in room light** | **Nystagmus with fixation blocked** | **SVV** | **HIT** |
| --- | --- | --- | --- | --- | --- | --- | --- |
| A | 2 days | dVM | Vertigo, headache | None | None | 4.7°, R | Negative |
| E | 2 days | pVM | Vertigo | None | None | 3.0°, L | Negative |
| C | 2 hours | pVM | Vertigo | None | Downbeat PN | 2.3°, L | Negative |
| B | 7 hours | dVM | Vertigo, headache, photophobia, phonophobia, motion sickness | None | None | 2.0°, L | Negative |
| F | 1 day | dVM | Vertigo, headache | None | Right-beating HSN | 2.0°, L | Negative |
| F | 1 day | pVM | Vertigo, menstrual dizziness, motion sickness | None | None | 1.7°, L | Negative |
| B | 1 day | dVM | Vertigo, headache | None | None | 1.3°, R | Negative |
| A | 2 days | dVM | Vertigo, headache | None | Right-beating HSN | 1.0°, L | Negative |
| C | 5 hours | dVM | Vertigo, headache, phonophobia, motion sickness | None | None | 1.0°, L | Negative |
| B | 2 days | pVM | Vertigo | None | None | 1.0°, R | Negative |
| F | 2 days | pVM | Vertigo | None | None | 1.0°, R | Negative |
| B | 2 days | dVM | Vertigo, headache | None | Downbeat SN | 1.0°, L | Negative |
| F | 6 hours | dVM | Vertigo, headache, visual aura, motion sickness | None | None | 0.7°, L | Negative |
| A | 2 days | pVM | Vertigo, motion sickness | None | None | 0.7°, L | Negative |
| F | 2 days | pVM | Vertigo, photophobia | None | Left-beating SN, downbeat PN | 0.7°, R | Negative |
| A | 9 hours | dVM | Vertigo, headache | None | Right-beating SN, PN | 0.7°, L | Negative |
| B | 2 days | pVM | Vertigo, motion sickness | None | None | 0.3°, L | Negative |
| C | 2 days | pVM | Vertigo, visual aura | None | Downbeat HSN, PN | 0.3°, L | Negative |
| F | 2 days | pVM | Vertigo | None | None | 0.3°, R | Negative |
| A | 2 days | dVM | Vertigo, headache, visual aura, motion sickness | None | Downbeat SN, PN | 0° | Negative |
| C | 1 day | dVM | Vertigo, headache, motion sickness | None | None | 0° | Negative |
| F | 1 day | pVM | Vertigo | None | Downbeat PN | 0° | Negative |
| D | 2 days | dVM | Vertigo, headache | None | Direction-changing PN | 0° | Negative |
| E | 10 hours | dVM | Vertigo, headache, photophobia, phonophonia, motion sickness | None | Downbeat HSN | 0° | Negative |
| C | 7 hours | dVM | Vertigo, headache, motion sickness | None | Left-beating PN | 0° | Negative |
| C | 4 hours | dVM | Vertigo, headache, photophobia | None | Left-beating HSN | 0° | Negative |
| C | 1 day | pVM | Vertigo | None | Left-beating HSN | 0° | Negative |
| A | 4 hours | dVM | Vertigo, headache | None | None | 0° | Negative |
| F | 2 days | dVM | Vertigo, headache, photophobia, phonophobia, motion sickness | None | None | 0° | Negative |
| F | 11 hours | dVM | Vertigo, headache, motion sickness | None | None | 0° | Negative |
| B | 1 day | pVM | Vertigo, motion sickness | None | None | 0° | Negative |
| B | 2 days | pVM | Vertigo | None | None | 0° | Negative |
| C | 8 hours | dVM | Vertigo, headache, photophobia | None | None | 0° | Negative |
| B | 4 hours | pVM | Vertigo | None | Left-beating HSN, PN | 0° | Negative |
| C | 2 days | dVM | Vertigo, headache, motion sickness | None | None | 0° | Negative |
| C | 2 days | dVM | Vertigo, headache | None | Left-beating HSN, direction-changing PN | 0° | Negative |

SVV, subjective visual vertical; HIT, head impulse test; dVM, “definite” vestibular migraine according to the diagnostic criteria of vestibular migraine (please see **Additional Table 1**); pVM, probable vestibular migraine according to the diagnostic criteria of probable vestibular migraine (please see **Additional Table 1**); PN, positional nystagmus; HSN, head-shaking nystagmus; SN, spontaneous nystagmus; Age group, A 30-41 years; B 42-51 years; C 51-60 years; D, 61-71 years; E, >72 years; F, <29 years.
