# Supplementary material for: The bucket test differentiates patients with MRI confirmed brainstem/cerebellar lesions from patients having migraine and dizziness alone
Source: BMC Neurol. 2019 Sep 3;19:219. doi: 10.1186/s12883-019-1442-z (PMC6720090; doi:10.1186/s12883-019-1442-z)
Supplement: Supplementary file 5 — Table S4. The results of subjective visual vertical for healthy controls matching the age and sex in CVD. (DOCX 15 kb) [file 12883_2019_1442_MOESM5_ESM.docx]

**Additional Table 4.** The results of subjective visual vertical for healthy controls matching the age and sex in CVD

| **Case** | **Age Group** | **SVV** |
| --- | --- | --- |
| 1 | D | 0.3°, L |
| 2 | B | 2.0°, L |
| 3 | A | 0.3°, R |
| 4 | D | 0° |
| 5 | B | 1.0°, R |
| 6 | D | 0° |
| 7 | D | 1.3°, R |
| 8 | A | 0° |
| 9 | E | 0.7°, L |
| 10 | A | 0.7°, L |
| 11 | E | 0° |
| 12 | E | 1.7°, L |
| 13 | B | 0.7°, R |
| 14 | C | 2.0°, L |
| 15 | B | 0.3°, L |
| 16 | B | 1.0°, L |
| 17 | B | 0° |
| 18 | D | 0.7°, L |
| 19 | E | 1.7°, R |
| 20 | C | 1.7°, L |
| 21 | D | 4.0°, L |
| 22 | D | 0° |
| 23 | A | 0.7°, R |
| 24 | C | 0° |
| 25 | C | 0° |
| 26 | D | 0.3°, R |
| 27 | E | 2.3°, L |

Age group, A 30-41 years; B 42-51 years; C 51-60 years; D, 61-71 years; E, >72 years; F, <29 years.
